# Autologous Fat Grafting for Craniofacial Reconstruction in Oncologic Patients

**DOI:** 10.3390/medicina55100655

**Published:** 2019-09-29

**Authors:** Cristian Ilie Drochioi, Daniela Sulea, Daniel Timofte, Veronica Mocanu, Eugenia Popescu, Victor Vlad Costan

**Affiliations:** 1 Department of Surgery, Oral and Maxillofacial Surgery, Faculty of Dental Medicine, Grigore T. Popa University of Medicine and Pharmacy, Iasi 700115, Romania; cristian_dro@yahoo.com (C.I.D.); jenypopescu@yahoo.com (E.P.); victorcostan@gmail.com (V.V.C.); 2 Department of Surgery, Faculty of Medicine, Grigore T. Popa University of Medicine and Pharmacy, Iasi 700115, Romania; 3 Department of Pathophysiology, Faculty of Medicine, Grigore T. Popa University of Medicine and Pharmacy, Iasi 700115, Romania; veronica.mocanu@umfiasi.ro

**Keywords:** fat graft, lipofilling, reconstruction, adipocyte, stem cell, cancer

## Abstract

Due to the anatomical and functional complexity of the region, craniofacial tumor removal requires some of the most challenging surgical approaches, often complemented with advanced chemo-radiotherapy techniques. However, these modern therapies often lead to sequelae that can drastically reduce the quality of life for the surviving patients. Recent advances in the field of regenerative medicine opened new avenues for craniofacial reconstruction following head and neck cancer treatment. One of the most promising recent strategies relies on the use of autologous fat transplant. In this mini review, we briefly present some of the fat’s biological properties that make it an ideal tissue for craniofacial reconstruction following cancer treatment. We then outline the recent advances that led to a better understanding of the detailed anatomy of the craniofacial fat depots. Furthermore, we provide a succinct review of the methods used for fat harvesting, processing and engrafting in the craniofacial area after head and neck tumor removal, discussing their main applications, advantages and limitations.

## 1. Introduction

The head and the neck are among the most complex anatomical regions of the human body. They are critical for our ability to perform essential activities for our survival, such as feeding, drinking, breathing and speaking. Additionally, the face is crucial for human identity and expressing emotion, consciously or unconsciously. Consequently, damages to the head and neck impinge the patient’s ability to breathe, chew, swallow, or talk and change his/her appearance, with significant adverse impact on the psyche.

Head and neck cancers broadly include carcinomas arising from the mucosal epithelia of the head and neck region, as well as various cell types of salivary glands and the thyroid. Among them, head and neck squamous cell carcinoma (HNSCC) is the sixth leading cancer by incidence worldwide, with approximately 600,000 new cases and 40–50% mortality [1]. Salivary gland cancers are relatively uncommon, accounting for only 1–6% of all neoplasms in this region [2]. The most important risk factors for head and neck cancers are tobacco smoking and alcohol consumption, with a synergistic carcinogenic effect [3]. A subgroup of HNSCCs, particularly those of the oropharynx, is caused by sexually transmitted infections with high-risk types of human papillomaviruses (HPV), especially HPV type 16 [4]. 

Treatment options for head and neck cancers include surgery, radiation therapy, chemotherapy, and targeted therapy, frequently applied in combinations. Surgery and radiotherapy for head and neck cancers often lead to significant morphological sequelae, such as contour defects and irregularities, asymmetries, and atrophic skin, even in patients for whom microsurgical reconstruction has been performed. Consequently, secondary surgical correction of these defects is often required. Autologous fat transplant in the craniofacial area was first applied in 1893 by Neuber, who filled a depressed facial defect using small pearls of fat taken from the arm of the same patient [5]. However, this method only gained significant interest owing to the work performed in 1990s by Coleman et al. [6,7], who standardized the methods for fat harvesting and engrafting. The fat is the closest to the ideal tissue filler for craniofacial reconstructive surgery because it is readily available, easily obtainable, repeatable, inexpensive, versatile, and biocompatible. Autologous fat transplant has become considerably popular in the recent years, due to new techniques that are minimally invasive, safer and deliver more predictable outcomes. Despite these ideal characteristics and technical advances, the published scientific literature in this area remains scarce and highly fragmented. This mini review aims to provide a succinct overview of the key recent advances in the field of autologous fat transplant for craniofacial reconstruction in head and neck oncologic patients.

## 2. Fat Biology and Its Relevance to Regenerative Medicine

The fat, also known as adipose tissue, is a master regulator of energy balance and nutritional homeostasis. However, the fat also plays many other important functions, from thermogenesis and insulation, to influencing the activities of other tissues and organs via a diverse class of molecules collectively termed adipokines, and to mechanically protecting delicate organs such as the eyes, which are wrapped around in a cushion of fat [8]. 

In humans, as well as in all other mammals, two principal types of adipose tissue exist, white (WAT) and brown (BAT). Brown adipocytes contain multilocular lipid droplets and high numbers of mitochondria, and primarily function to dissipate stored energy in the form of heat, through the actions of UCP1 (Uncoupling Protein-1), located in the inner mitochondrial membranes of these cells [9]. The main BAT depots reside in the paracervical and supraclavicular areas, as documented with PET (positron-emission tomography) techniques [10]. However, the vast majority of adipose tissue in humans is WAT, which is primarily comprised of large adipocytes, with a single lipid droplet and markedly fewer mitochondria than brown adipocytes. WAT is organized into two main anatomical compartments, identified as subcutaneous adipose tissue (SAT, such as the abdominal and the gluteofemoral fat depots) and visceral adipose tissue (VAT, such as the omental, mesenteric, retroperitoneal, and pericardial fat depots) [11]. More recently, cells possessing characteristics of both brown and white adipocytes (known as ‘beige’ or ‘brite’ adipocytes) have also been described [12]. Given that only WAT is being used for autologous fat transplants, we will only refer to this type of fat henceforward. 

During development, WAT originates mainly from the mesoderm [13], with the notable exception of the facial fat, which develops from the neural crest [14]. The recently developed ‘AdipoChaser’ mouse model [15] allowed detailed understanding of the developmental timing of various fat depots. This model revealed that SAT adipocyte commitment and differentiation occurs at a stage equivalent to the second trimester of human pregnancy and that the number of SAT adipocytes remains very stable in the postnatal life. In contrast, VAT adipocytes preferentially differentiate after birth [15]. 

**Figure 1:**
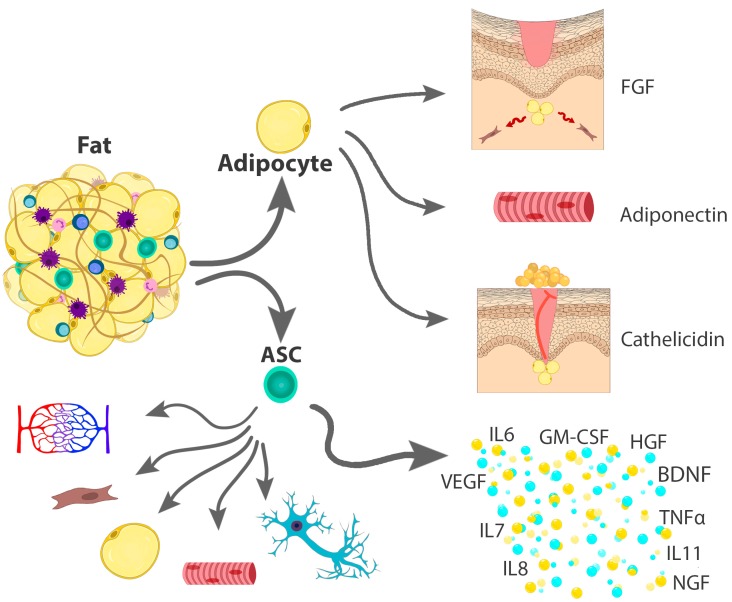
Biological roles of adipocytes and adipose-derived stem cells (ASCs) in regenerative medicine. The fat is composed of mature adipocytes and a variety of stroma vascular cells (SVCs), all embedded into the extracellular matrix. The mature adipocytes promote wound healing by attracting fibroblasts through FGF (fibroblast growth factor) and preventing skin infection with *S. aureus* by secretion of cathelicidin. They also promote muscle regeneration via adiponectin. ASCs have the potential to differentiate into multiple cell types, such as adipocytes, fibroblasts, neurons, muscle or endothelial cells. They also secrete a large variety of growth factors such as hepatocyte growth factor (HGF), vascular endothelial growth factor (VEGF), brain-derived neurotrophic factor (BDNF), nerve growth factor (NGF), granulocyte-macrophage colony-stimulating factor (GM-CSF), interleukins (IL) 6, 7, 8 and 11, tumor necrosis factor alpha (TNFα), and a variety of adipokines, all of which have trophic effects of many cell types.

The adipose tissue is highly heterogeneous, containing mature adipocytes, stromal-vascular cells (SVCs), blood vessels, lymph nodes and nerves, all imbedded in the extracellular matrix containing different types of collagen [8]. Although the mature adipocytes occupy over 90% of the entire fat volume, they represent only around 20–40% of all fat cells [8]. In addition to their value as a ‘filler’ in the reconstructive surgery, mature adipocytes have been shown to actively promote wound healing (Figure 1), being required for fibroblast recruitment during dermal reconstruction [16], as well as for protecting against invasive *Staphylococcus aureus* skin infections through the production of the cathelicidin, an effective antimicrobial peptide [17]. Additionally, among the many adipokines secreted by the mature adipocytes, adiponectin has emerged as a potent tissue-regenerating hormone, particularly for the skeletal muscle [18]. SVCs represent 60–80% of all cells that form the fat, of which more than half are leukocytes (macrophages, neutrophils, mast cells, eosinophils, B lymphocytes, and various classes of T lymphocytes). The other cells that form SVCs are adipose-derived stem cells (ASCs), pre-adipocytes, endothelial cells, pericytes, and fibroblasts (Figure 1). 

ASCs have recently received particular attention in the field of regenerative medicine because they can be easily isolated, while providing higher yields upon the processing of adipose tissue compared to other stem cell populations and sources. According to a common statement by the International Federation for Adipose Therapeutics (IFATS) and the International Society for Cellular Therapy (ISCT) in 2013, ASCs are identified phenotypically upon performing flow cytometry, as cells negative for leukocyte markers (cluster of differentiation (CD) 45−), negative for endothelial cell markers (CD31−) and positive for common mesenchymal stem cell markers (CD73+, CD90+, CD105+, CD44+). However, contrary to other mesenchymal stem cells, ASCs are also classified as CD36+ and CD106− cells [19]. Similar to other stem cell populations, the value of ASCs for regenerative medicine is intimately related to their differentiation capabilities into multiple cell types. Additionally, ASCs exhibit immunomodulatory, as well as trophic effects on a variety of endogenous cells/tissues. These properties are closely related to the ASCs’ secretome [20], which consists of molecules such growth factors, interleukins and a variety of adipokines (Figure 1).

## 3. The Anatomy of Facial and Neck Fat

Facial fat consists of superficial and deep depots that are separated from each other by the superficial musculo-aponeurotic system [21,22]. The superficial fat depots, characterized using dye injections (such as methylene blue) in cadavers, followed by dissections or by contrast-enhanced computed tomographic scans followed by three-dimensional reconstructions, are numerous and distribute broadly under the facial skin, being separated by natural septal boundaries. These depots have been described in great detail in several recent comprehensive reviews [23,24]. The deep facial fat depots have been characterized using both computed tomographic and magnetic resonance imaging techniques aided by injections with colored contrast-enhancing substances, followed by three-dimensional reconstruction [24,25]. Similar with the face, the neck also contains distinct superficial and deep fat compartments [26,27].

Each of these fat pads has distinct morphological and metabolic characteristics. For example, the average adipocyte size of the nasolabial fat is significantly larger than that of the deep medial cheek fat [28]. Additionally, the facial fat depots have different collagen and hyaluronic acid composition in their extracellular matrix [21]. These differences provide unique and specific mechanical and histochemical characteristics to each of the respective compartments, properties that are relevant for facial fat grafting.

One study focused on the histologic analysis of the different fat compartments of the face. The authors concluded that the configuration of adipocytes found in the facial fat deposits is not only related to the function of the fat pads, but also to their embryologic origin [29]. The same study distinguishes three types of white adipose tissue—deposit, fibrous or structural—in accordance with the classification of Sbarbati et al. [30]. It was suggested that the histologic characteristics of the adipose tissue in the receiving and donor areas should be considered when performing autologous fat transplant for regenerative purposes. In this regard, the site for adipose tissue harvesting should not be chosen randomly but should be chosen considering the structural characteristics of the regional fat deposits [30].

From a histologic standpoint, Bichat’s fat pad showed high similarity with visceral fat tissue [29]. Conti et al. [31] have shown the increased regenerative potential of Bichat’s fat pad that could be used as an easily accessible alternative fat harvesting site for defect reconstruction using lipofilling, consecutive to proper processing. 

## 4. Autologous Fat Transplantation Procedure

The key steps in the autologous fat procedure include fat harvesting, fat processing and fat engrafting. Each of these steps has an important impact on the craniofacial fat graft viability and the overall outcome in oncologic patients.

### 4.1. Fat Harvesting

The lower abdomen, the buttocks, the lateral femoral region and the interior side of the upper thighs are all ideal sites for fat harvesting, due to the distribution of human adipose tissues and relatively large depots. In patients with very low percentage of body fat, ultrasound examination is helpful to determine the thickness and depth of adipose depots before fat harvesting [32]. Despite reported biological differences between different donor sites, such as size of adipocytes, enzyme activity, density of ASCs or proportion of connective tissue, there is no convincing evidence to suggest that the donor site has a significant impact on the overall outcome of the autologous fat transplant [33]. 

Fat harvesting is performed under general anesthesia or regional block, using an array of techniques and methodologies that fall under three main categories: surgical excision, vacuum liposuction and manual aspiration through cannula [34,35]. The surgical excision techniques involve less damage to the adipocytes; however, they are more likely to leave cicatrices at the donor site and, hence, are less popular. On the contrary, vacuum aspiration techniques, owing to the negative pressure used, are more traumatic for the tissue and may cause destruction of the cellular structures in up to 90% of all adipocytes yielded [36]. However, new methods such as water-assisted liposuction, in which a dual-purpose cannula that emits pulsating, fan-shaped jets of tumescent solution and simultaneously suctions fatty tissue and the instilled fluid through a separate channel within the cannula into an integrated suction unit, greatly improve fat graft survival [37]. These methods are successfully used in cases when larger amounts of fat are required, such as in breast lipofilling. 

Due to the low or moderate amount of fat required for craniofacial transplants, methods based on manual aspiration through cannula are most widely used. There are many types of cannulas used, with different diameters, number of holes and hole sizes. According to several national surveys, Coleman’s technique is the most routinely used method for fat harvesting in autologous craniofacial fat transplants. It consists in injecting the donor site with local anesthetic (a combination of 0.5% lidocaine and epinephrine 1:200,000) and the fat being harvested using a 3-mm blunt edge, two-hole cannula connected to a 10-cm^3^ syringe, by manually withdrawing the plunger under a mild negative pressure [7,38].

### 4.2. Fat Processing

After harvesting, processing techniques such as sedimentation, filtering, washing, and centrifugation are used, in order to remove nonviable components of the aspirate. Multiple protocols for adipose tissue collection and processing have been developed [39,40,41]. There is no consensus for an optimal method of fat graft processing. However, Coleman’s method, consisting in spinning the aspirate at 3000 rpm (1500× *g*) for 3 min, which separates the aspirate into a top oil layer originating from burst adipocytes, a middle layer of usable fat tissue, and a bottom layer of blood and tumescent solution, has been shown to constantly produce grafts of good quality and, consequently, has been integrated in many autologous fat transplant protocols [36,38]. Lower centrifugal forces of 1300 rpm (250× *g*) for 5 min may improve even more fat graft viability [42]. Additionally, De Francesco et al. [43] stated the differences between the layers resulted after fat centrifugation. They demonstrated that in the middle high-density layer there is an optimal source of stem cells that can be used for regenerative purposes [43].

Additional processing methods have been developed, aimed to improve fat graft uptake, reduce graft resorption rate (estimated to be between 20% and 80%), decrease the adverse effects (e.g., fibrosis, pseudocyst formation, and calcification) and produce more predictable and longer-lasting results than the lipofilling alone. These include the addition of bio-enhancers (such as insulin, insulin growth factor 1 or type I collagen) to support adipogenesis, and the use of three-dimensional synthetic scaffolds [44]. However, the most widely used method is the so-called cell-assisted lipotransfer (CAL), first introduced in 2006, which consists in the transplantation of aspirated fat enriched with ASCs contained in the stromal vascular fraction obtained after enzymatic digestion of fat or after cell culture [45]. Nevertheless, because potent stem cells can also stimulate oncogenesis, the safety of this method is still debated for oncologic patients [46].

The enzymatic method is the most indicated for isolating the stromal vascular fraction, but its use is limited in Europe due to restrictions imposed by regulatory issues referring to enzymatic procedures [47,48]. The enzyme-free, mechanical methods of obtaining the stromal vascular fraction (Rigenera^®^, Human Brain Wave HBW, Turin, Italy; Lipogems^®^, Lipogems International SpA, Milan, Italy; Body-jet^®^, Human Med AG, Schwerin, Germany) offer a useful alternative, involving minimal manipulation of the cells, in accordance with the regulatory European directives 120/2009, 23/2004 and the annex I of the directive 1394/2007 [47,48,49,50,51]. These methods have been proven just as effective in obtaining fat tissue enriched in quality adipose-derived mesenchymal stem cells [47,48].

### 4.3. Fat Engrafting

The method used for fat placement is key for the short-term, as well as the long-term success of the transplant. Most frequently, a cannula is used to create multiple “tunnels” in which small fat parcels are deposited only during the withdrawal of the cannula, thus obtaining a large contact surface area between the fat and the local capillaries. Coleman’s technique performs microinjections through multiple access sites, using a 17-gauge blunt cannula with one hole at the distal end and connected to a 1-mL syringe [52]. Other sizes, tip shapes, lengths and curves may be suitable for particular areas such as the peri-orbital region [34]. Delivering fat directly into the intrinsic facial musculo-aponeurotic system with blunt-tipped cannulas has been shown to reduce the frequency of fat resorption, which is more likely to occur for the fat transplanted into mobile areas of the face, such as the lips, compared to the less mobile areas such as the lateral cheek [34].

## 5. Applications of Autologous Fat Grafting for Craniofacial Reconstruction Following Cancer Treatment

The advances in the head and neck cancer treatment have improved overall survival significantly, leading to an ever-increasing interest in the quality of life after treatment. An important component of this management is the correction of the defects caused by the surgery and radiotherapy [53,54]. These defects depend on the type of tumor and its location, and are generally classified into two main categories: esthetic and functional. The esthetic defects, which often appear even in patients in whom microsurgical reconstruction has been performed, are represented by contour defects and irregularities, asymmetries, and atrophic skin. Functional defects consist of reduced neck mobility, dysphagia, aspiration, the inability to swallow, the Frey syndrome following parotidectomy, and diminished phonation following laryngeal cancer treatment. Both categories of defects can benefit from autologous fat grafting (Table 1) [53,54,55,56,57,58,59,60,61,62,63].

There is controversy regarding the use of autologous fat grafting for reconstructing defects following oncologic surgery. This is due to concern related to the possible stimulation of recurrence onset due to the regenerative properties of the lipoaspirate. However, up to date there is no conclusive evidence in this regard. Furthermore, studies performed so far on lipostructure breast reconstruction in oncologic patients did not identify an increased incidence of local recurrence [64,65,66,67]. Some authors describe measures for safety by performing accurate imaging before and after lipografting procedures, with the biopsy of suspicious lesions, as well as increasing the time interval between the tumor removal and lipofilling in aggressive, extended malignancies [65,66,67]. Nevertheless, more multicenter prospective studies with long follow-up periods are necessary to reinforce the results, particularly concerning head and neck cancer reconstruction.

### 5.1. Autologous Fat Grafting for Healing Irradiated Head and Neck Skin Damage

Irradiation doses >50 Gy, typically used for the HNSCC therapy, have been shown to reduce the density of the subcutaneous microvasculature network, with consequent striatal muscle dystrophy, areas of adiponecrosis and fibrosis underneath the ischemic skin [56]. These observations have been confirmed in a study performed on mice exposed to external beam radiation on the scalp area, followed four weeks later by injections of human fat grafts in the subcutaneous plan of the irradiated skin. Similar to what was observed in clinical cases, fat grafting attenuated dermal collagen deposition and vessel depletion, characteristic to radiation fibrosis. Although the retention rate of the transplanted fat was lower in previously irradiated mice compared to controls, the quality of the retained fat between the two groups was comparable [68].

The first retrospective study that used autologous fat grafting as an alternative to conventional techniques, such as flaps or microsurgical techniques, for correcting esthetic defects after radiotherapy, was published in 2009 [56]. In this small study, eleven patients received fat harvested using Coleman’s technique, as described above, with the notable difference of skipping the centrifugation step before engrafting. For nine patients with greater than two years follow-up, both functional and aesthetic improvements were observed. Notably, histological follow-up was performed in six of the patients, which revealed an improvement of the vascular network density, reduced fibrosis and absence of necrotic areas. However, six patients required fat reinjection three months after the first treatment, due to fat resorption, which led to volume loss in proportion of 20–40% [56]. A prospective study published in 2017 [60] included ten patients that had undergone surgery and received ≥60 Gy of external radiotherapy followed by autologous fat transfer after at least three years of complete remission. The fat grafts led to esthetic and functional enhancement in most patients, with improvement of the skin blood supply and softness and better neck mobility and mouth opening in those patients that received the injections in the cervical area and lips, respectively. Fat resorption, in proportion of 25–50%, was observed in all patients [60]. 

In the largest study published so far on autologous fat transplants performed in oncologic head and neck patients, 83 cases out of the total 116 included had received radiation therapy before the fat grafting [63]. The mean time span between the end of the radio-surgical therapy and the first fat graft was approximately three years, with an additional three years of follow-up after the grafting. This study demonstrated the safety of the fat grafting, with low rate of procedure-related complications (~5%), very rare occurrence of fat necrosis (<1%) and no significant impact on the risk for local recurrence. Additionally, the long-lasting esthetic outcomes, subjectively scored by plastic surgeons and laypersons approximately two years after the procedure, were significantly ameliorated [63].

The quality of the skin in irradiated areas is increased following lipotransfer. This may be explained by changes in the structure of the local tissues. In this regard, one study found an improvement in the pattern of collagen and elastic dermis fibers following autologous fat transfer with expanded mesenchymal stem cells [69]. Another study showed an increased tissue hydration following autologous fat transfer in the region of radiotherapy damaged tissue, alongside the formation of new blood vessels and increased local regeneration [70]. The authors underlined the possible usefulness of lipostructure for the treatment of other microangiopathic conditions [70].

### 5.2. Autologous Fat Grafting for Craniofacial Contour Correction Following Flap Reconstruction

Although regional flaps provide acceptable coverage of the defects left after head and neck cancer surgery, they often have inadequate volumes compared to the normal tissues, thus requiring esthetic contour correction. This situation is particularly common when cervical flaps (that have relatively little subcutaneous fat) are used to reconstruct defects following tumor removal in the cheek area [71]. The first example of this application for autologous fat grafting was published in 2003 in a small series of six patients who underwent one or two transplants each [55]. Five of these patients had a good esthetic outcome, based on a subjective scoring. 

**Figure 2:**
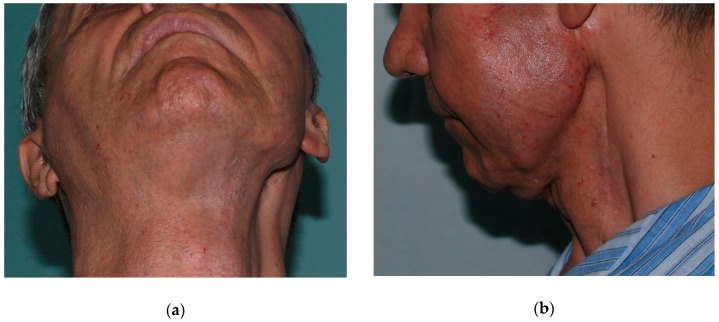
Patient with facial asymmetry and sunken aspect of the left parotid region due to an operated, irradiated, adenoid cystic carcinoma of the left parotid gland: (**a**) Frontal view; (**b**) Profile view.

**Figure 3:**
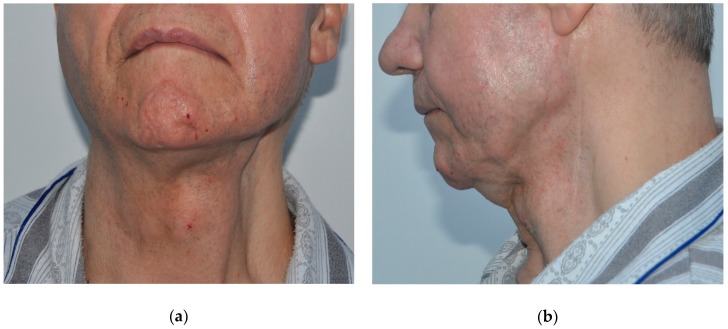
Clinical aspect of the same patient one year after two lipostructure procedures in the left parotid region, showing improved facial symmetry and contour of the parotid area. The first autologous fat grafting procedure was performed 1 year after radiotherapy, followed by another session after 6 months. (**a**) Frontal view; (**b**) Profile view.

Several studies have also shown good cosmetic results following fat grafting for improving the facial contour following parotidectomies due to benign or malign tumor resections [72] (Figure 2 and Figure 3). An additional benefit of fat grafting in these patients is the prevention of Frey’s syndrome, i.e., redness and sweating on the cheek, temporal and retroauricular regions, in response to gustatory stimuli. Furthermore, fat grafting has also been used for correcting more subtle defects (such as deformities and depressions) left after lip reconstruction with a nasiolabial flap following malignant or benign tumor removal [59].

### 5.3. Autologous Fat Grafting for the Correction of Functional Defects

Structural fat grafting has been used in recent years for treatment of several types of functional defects following craniofacial cancer treatment. One such application is the vocal folds augmentation in cases with scars left after surgery or radiotherapy. For example, a recent study of fat grafting in 79 dysphonic patients, who included ten with surgery for malign or benign tumors, led to voice improvement in all patients [61]. Another published application was the correction of insufficient neoglottal closure following supracricoid laryngectomy with cricohyoidoepiglottopexy for laryngeal cancers [58]. In both patients described in this study, suffering from chronic aspiration, the autologous fat was harvested from the buccal fat pad and injected into the widest plane of the neoglottis under direct laryngoscopy, leading to improvement of swallowing. However, fat resorption occurred in both cases and only approximately one-third of the injected fat persisted at 6 months after augmentation [62]. In another case series of seven HNSCC patients suffering from chronic oropharyngeal dysfunction, as result of volume loss or muscle atrophy of the tongue or pharyngeal musculature following treatment with surgery and/or chemoradiotherapy, autologous lipofilling was shown to be safe and led to swallowing improvement in four cases [62]. The case series published by Ducic et al [55] also included two patients with patients with velopharyngeal insufficiency and one with bi-maxillary swing following oncologic oropharyngeal resection. Significant improvement of both hypernasality and nasopharyngeal reflux for food was reported in each case [55]. Finally, functional improvement following autologous lipofilling in the base of the tongue was reported in the case of a patient treated with radiation therapy for a nasopharyngeal carcinoma who presented with severe postradiation dysphagia [57]. The positive outcomes of lipofilling for improving the sequelae of tracheostomy, velo-pharyngeal insufficiency and closing pharyngo-cutaneous fistulae are outlined by Mazolla et al. [73].

## 6. Conclusions

The studies published so far on autologous fat transfer following craniofacial cancer treatment seem to suggest that this technique may provide an effective treatment for a large range of esthetic and functional sequelae. However, with a few exceptions, all studies published so far include relatively low number of patients, and there are notable differences regarding the interval of time that passed between cancer treatment and the autologous fat transplant, as well as the details of the techniques used for fat harvesting and grafting and the outcome parameters measured. These differences led to a body of evidence that is highly fragmented and difficult to analyze and they highlight the need for well-designed, large clinical trials that could lead to the establishment of optimal guidelines. Until then, some of the recently published systematic reviews and meta-analyses [63,74] may pave the way in the continuous search for the optimal autologous fat grafting methods for oncologic head and neck reconstruction that provide the best long-term outcomes with the lowest rates of complications. Future preclinical studies aimed at better understanding the biology and differences between various craniofacial fat pads, as well as the potential safety risks related to the use of ASCs following cancer treatment, may lead to additional refinements of the autologous fat grafting methods used for craniofacial reconstruction in oncologic patients.

## Figures and Tables

**Table 1:** Studies describing applications of autologous fat grafting for craniofacial reconstruction in oncologic patients [53,54,55,56,57,58,59,60,61,62,63].

Study (Reference)	Number of Oncologic Patients	Time from End of Cancer Treatment	Technique of Fat Harvesting	Volume of Fat Injected	Time of Follow Up	% Fat Resorption
**Ducic Y et al. 2003 [55]**	23	>1 year	Coleman	n/a	up to 5 years	n/a
**Phulpin B et al. 2009 [56]**	11	n/a	Coleman	20 to 45 mL	2 to 88 months (mean, 39.9 months)	20–40%
**Navach V et al. 2011 [57]**	1	>10 years	Coleman	5 mL	3 months	n/a
**Costan VV et al. 2012 [53]**	8	n/a	Coleman	10 to 45 mL	n/a	n/a
**Nakayama M et al. 2013 [58]**	2	3–5 years	Manual surgery	0.8 mL or 0.7 mL	6 months	67–70%
**Drochioi C et al. 2015 [54]**	18	n/a	Coleman	10 to 45 mL	n/a	n/a
**Vitagliano T et al. 2016 [59]**	10	12 months	Coleman	10 mL	n/a	n/a
**Gutiérrez Santamaría J et al. 2017 [60]**	12	12 months	Coleman	5 to 70 mL	n/a	25–50%
**Cantarella G et al. 2017 [61]**	10	n/a	Coleman	0.2–0.4 mL	3 to 36 months	n/a
**Kraaijenga SAC et al. 2017 [62]**	7	~12 months	Coleman	11 to 34.5 mL	6 to 8 months	n/a
**Karmali RJ et al. 2018 [63]**	116	40.5 ± 24.3 months	50% Coleman; 39% Manual; 8.9% Cytori; 2.1% Revolve	24.8 ± 20.2 mL	35.8 ± 23.1 months	n/a
